# Comparison of oral microbiome profiles in stimulated and unstimulated saliva, tongue, and mouth-rinsed water

**DOI:** 10.1038/s41598-019-52445-6

**Published:** 2019-11-06

**Authors:** Ryutaro Jo, Yuichiro Nishimoto, Kouta Umezawa, Kazuma Yama, Yuto Aita, Yuko Ichiba, Shinnosuke Murakami, Yasushi Kakizawa, Takashi Kumagai, Takuji Yamada, Shinji Fukuda

**Affiliations:** 1 Oral Care Research Laboratories, Research and Development Headquarters, Lion Corporation. 7-2-1 Hirai, Edogawa-ku, Tokyo 132-0035 Japan; 2 Metabologenomics, Inc. 246-2 Mizukami, Kakuganji, Tsuruoka, Yamagata 997-0052 Japan; 3 Hiyoshi Oral Health Clinics. 2-1-16 Hiyoshi-cho, Sakata, Yamagata 998-0037 Japan; 4 Advanced Analytical Science Research Laboratories, Research and Development Headquarters, Lion Corporation. 7-2-1 Hirai, Edogawa-ku, Tokyo 132-0035 Japan; 5 Institute for Advanced Biosciences, Keio University, 246-2 Mizukami, Kakuganji, Tsuruoka, Yamagata 997-0052 Japan; 6 Department of Life Science and Technology, Tokyo Institute of Technology, 2-12-1 Ookayama, Meguro, Tokyo 152-8550 Japan; 7 PRESTO, Japan Science and Technology Agency, 4-1-8 Honcho Kawaguchi, Saitama, 332-0012 Japan; 8 Intestinal Microbiota Project, Kanagawa Institute of Industrial Science and Technology, 3-25-13 Tonomachi, Kawasaki-ku, Kawasaki, Kanagawa 210-0821 Japan; 9 Transborder Medical Research Center, University of Tsukuba, 1-1-1 Tennodai, Tsukuba, Ibaraki 305-8575 Japan

**Keywords:** Microbiome, Oral microbiology

## Abstract

Epidemiological studies using saliva have revealed relationships between the oral microbiome and many oral and systemic diseases. However, when collecting from a large number of participants such as a large-scale cohort study, the time it takes to collect saliva can be a problem. Mouth-rinsed water, which is water that has been used to rinse the oral cavity, can be used as an alternative method for collecting saliva for oral microbiome analysis because it can be collected in a shorter time than saliva. The purpose of this study was to verify whether mouth-rinsed water is a suitable saliva substitute for analyzing the oral microbiome. We collected samples of mouth-rinsed water, stimulated saliva, unstimulated saliva, and tongue coating from 10 systemic healthy participants, and compared the microbial diversity and composition of the samples using next-generation sequencing of 16S rRNA-encoding genes. The results showed that the microbial diversity of mouth-rinsed water was similar to that of unstimulated and stimulated saliva, and significantly higher than that of tongue-coating samples. The microbial composition at the species level of mouth-rinsed water also showed a very high correlation with the composition of unstimulated and stimulated saliva. These results suggest that the mouth-rinsed water is a suitable collection method instead of saliva for oral microbiome analysis.

## Introduction

Following the development of genome analysis technology using next-generation sequencing, the relationships between human microbiota and disease are being clarified. Even in the oral cavity, it has become clear that an imbalanced microbiota can contribute to various oral diseases, such as periodontal disease, dental caries, and halitosis^1–5^. The microbial composition of saliva has also been reported to be associated with autoimmune diseases, such as inflammatory bowel disease, rheumatoid arthritis, and many other systemic diseases^6–8^. Therefore, the microbial composition of saliva has been proposed for use as a risk assessment measure for many oral and systemic diseases. Saliva collection usually takes about 1 to 5 minutes per person. Therefore, when collecting samples from a large number of participants such as large-scale cohort studies, a simpler alternative method that shortens the collection time is required. As an alternative to saliva collection, a solution obtained by mouth washing using a commercially available mouth rinse (containing 15% ethanol) can be used^9,10^, but since it contains ethanol, this may also cause problems such as irritation to the oral mucosa or acute alcohol intoxication due to accidental ingestion of child. The turbidity of mouth-rinsed water, which is a liquid obtained by rinsing the oral cavity with water, is reported to reflect both oral health state and malodor^11,12^. The microbiome of mouth-rinsed water is expected to be influenced by that of the saliva and the tongue; however, the similarity of these microbial profiles has not yet been investigated.

In this study, as a first step to show the validity of mouth-rinsed water as an alternative method to saliva collection for the analysis of oral microbiome, we collected mouth-rinsed water, stimulated saliva, unstimulated saliva, and tongue coating from 10 systemic healthy participants, and compared the microbial diversity and composition of these samples using next-generation sequencing of 16S rRNA-encoding genes. We clarified that the microbiome of mouth-rinsed water was similar to that of unstimulated and stimulated saliva from the same participant, and showed that collecting mouth-rinsed water is a valid alternative sampling method to saliva collection for analyzing the oral microbiome.

## Results

### Comparison of oral microbial diversity

**Figure 1:**
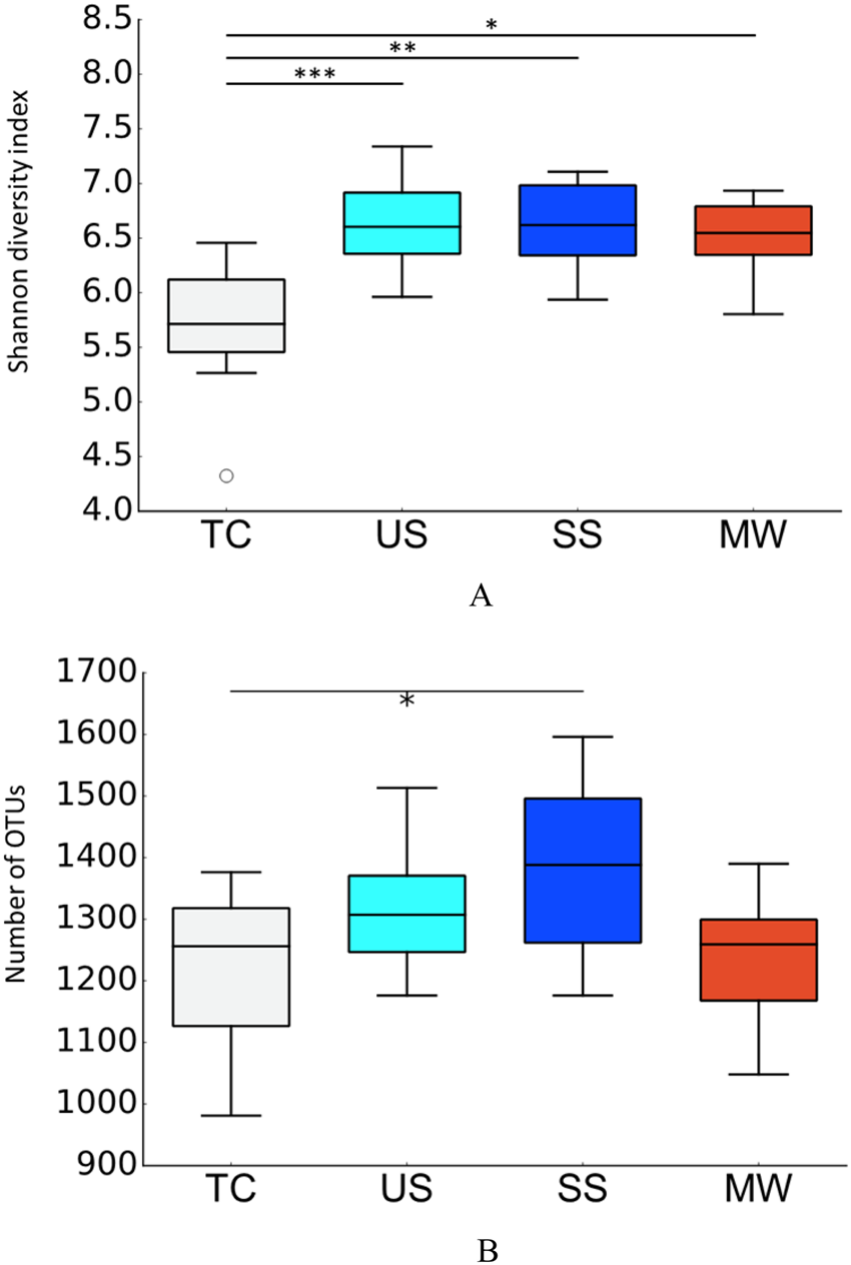
Alpha-diversity of oral microbiome in each sampling methods. Boxplots show the shannon diversity index (**A**) and the number of observed OTUs (**B**) of each sampling methods with the number of sequences rarefied to 10000 reads per sample. TC, Tongue coating; US, unstimulated saliva; SS, stimulated saliva; MW, mouth-rinsed water. Statistically significant differences are marked with asterisks (Nemenyi test, *p < 0.05 **p < 0.005 ***p < 0.0005).

Changing microbial diversity of the oral microbiome is an important indicator of many diseases, such as dental caries, periodontal disease, and diabetes^13–15^. Therefore, we firstly compared microbial diversity among the unstimulated saliva (US), stimulated saliva (SS), tongue coating (TC), and mouth-rinsed water (MW). Shannon’s diversity index, an indicator of microbial diversity, showed comparable results among US, SS, and MW samples but was significantly lower in TC samples (Fig. 1A). The median number of OTUs in MW was slightly lower compared with SS and US, although no statistically significant differences were detected between MW and SS or US, (SS–MW, *p* = 0.0581; US–MW, *p* = 0.264) (Fig. 1B).

### Comparison of oral microbiome profiles for each sampling method

**Figure 2:**
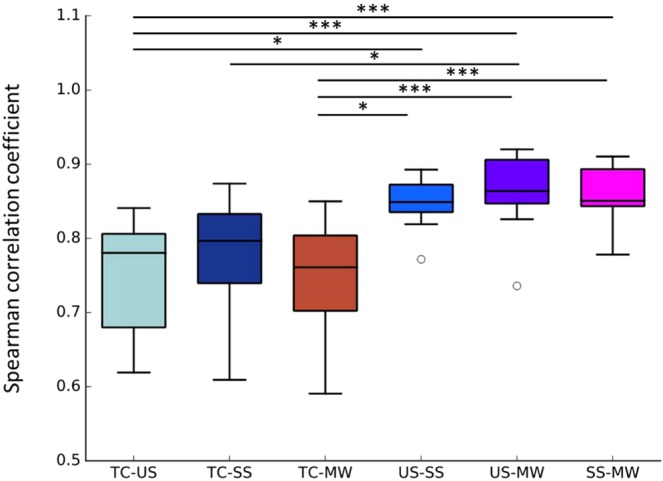
Spearman Rank Correlation between each sampling method. TC, Tongue coating; US, unstimulated saliva; SS, stimulated saliva; MW, mouth-rinsed water. Statistically significant differences are marked with asterisks (Nemenyi test, *p < 0.05 **p < 0.005 ***p < 0.0005).

**Figure 3:**
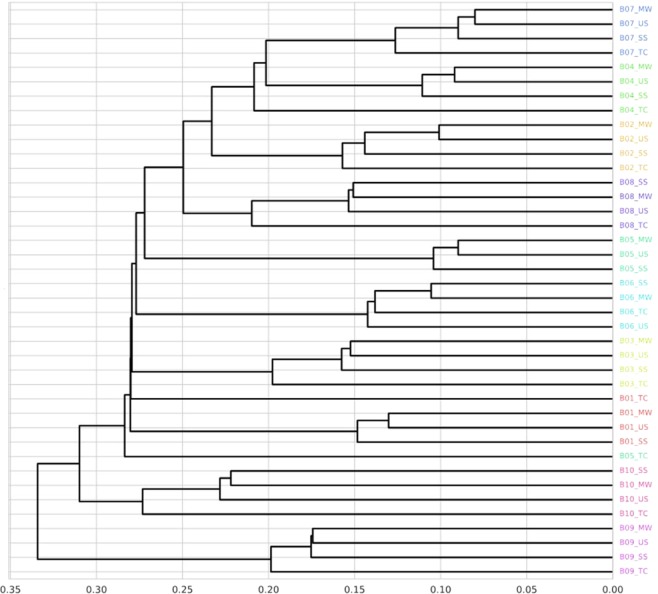
Cluster analysis based on Spearman Rank Correlation (single linkage method) . The color of the sample name is different for each subject. TC, Tongue coating; US, unstimulated saliva; SS, stimulated saliva; MW, mouth-rinsed water.

Subsequently, in order to compare the microbiome profiles of each sampling method, Spearman’s rank correlation coefficients at the species level between each sampling methods were compared. The results showed that the correlation coefficients between MW, SS, and US were significantly higher than that between MW and TC (Fig. 2, Supplementary Table S1). For all participants, the correlation coefficients between MW and SS/US were higher than those between MW and TC (Supplementary Table S2). The correlation coefficient between SS and US was also high (*r* = 0.846 ± 0.033). Similar results were obtained when using weighted or unweighted UniFrac distances (Supplementary Fig. S1). Furthermore, as a result of hierarchical clustering analysis, samples from the same individual were clustered except B05_TC (Fig. 3). Regardless of the status of dental caries or periodontal disease, MW, SS, and US were collected for all participants. Finally, we tested whether the relative abundance of individual bacteria changes depending on the collection method used. As a result, no genera were found to show significantly different relative abundances among the three sampling methods (Friedman test *q* < 0.10), whereas at the species level, eight species showed significant differences among the three sampling methods, and these species also showed significant differences between MW and US or SS (Table 1, Nemenyi post-hoc test *p* < 0.05).

**Table 1:** Bacterial species that showed significantly difference of relative abundance between mouth-rinsed water, and unstimulated saliva or stimulated saliva.

	Relative abundance			*p* or *q*-values		
	MW	US	SS	Friedman test	MW-US	MW-SS
*Capnocytophaga leadbetteri*	0.0018 ± 0.0016	0.0026 ± 0.0022	0.0018 ± 0.0015	0.075	0.003	N.S.
*Catonella morbi*	0.0004 ± 0.0004	0.0004 ± 0.0003	0.0001 ± 0.0002	0.075	N.S.	0.027
*Eikenella corrodens*	0.0004 ± 0.0006	0.0008 ± 0.0005	0.0003 ± 0.0002	0.090	0.020	N.S.
*Haemophilus haemolyticus*	0.0095 ± 0.0080	0.0031 ± 0.0024	0.0007 ± 0.0005	0.062	0.020	<0.001
*Haemophilus sp*. *HMT 908*	0.0086 ± 0.0129	0.0041 ± 0.0061	0.0022 ± 0.0043	0.062	N.S.	<0.001
*Streptococcus infantis clade 431*	0.0090 ± 0.0071	0.0113 ± 0.0081	0.0144 ± 0.0092	0.072	N.S.	<0.001
*Streptococcus sp*. *HMT 064*	0.0005 ± 0.0012	0.0007 ± 0.0018	0.0011 ± 0.0023	0.062	N.S.	0.005
*Veillonella rogosae*	0.0117 ± 0.0052	0.0118 ± 0.0059	0.0186 ± 0.0082	0.090	N.S.	0.005

Relative abundance showed mean ± standard deviation. Nemenyi test (p < 0.05) was conducted as a post-hoc test following Friedman test (q < 0.10). NS, not significant.

## Discussion

In this study, we showed that the microbial diversity and composition of MW was similar to US and SS but different from that of TC in 10 systemic healthy participants. These results indicate that MW sampling is a valid alternative method to saliva sampling for analyzing oral microbiome.

The microbial diversity of MW was not different from that of US and SS, indicating that it is possible to collect the entire microbiome from MW as well as saliva samples. On the other hand, the microbial diversity of TC was significantly lower than that obtained by the other three methods (Fig. 1). This result was consistent with that found in a previous study^16^.

The microbial composition of US was similar to MW and SS from the same individual, but B10 had a relatively lower similarity (US–MW: *r* = 0.736, US–SS: *r* = 0.771). The reason for this may be that the abundance of *Neisseria flava* in US was higher compared with SS and MW (the relative abundance of *Neisseria flava* was US, 0.255; SS, 0.094; and MW, 0.086). Therefore, the abundance of other minor species was changed.

As a result of hierarchical clustering, the same individuals were clustered. Therefore, it is considered that the characteristics of each participant’s oral condition are reflected even when the sample collection method is different. The reason why B05_TC did not cluster with the sample from the same participant in Fig. 3 is that the abundance of *Neisseria flavescens* in TC was higher compared with the other samples, and the abundance of some minor species in TC was considered to be low (the relative abundance of *Neisseria flavescens* was US, 0.162; SS, 0.157; MW, 0.209; and TC, 0.448). The relative abundance of most species also showed no significant differences among MW, US, and SS; however, some species, such as *Veillonella rogosae*, *Haemophilus haemolyticus*, and *Streptococcus infantis* showed significant differences. Further analysis in a large scale cohort study should be required for understanding the reason, but attention should be paid to these bacteria when comparing the results of salivary microbiome analysis even though there were no significant differences in the genera to which these bacteria belong.

It has been reported that the similarity of oral microbiome profile between US and SS were controversial^17,18^. In this study, the oral microbial diversity and composition of US and SS from the same participant were comparable. In the study showed the similar pattern of microbial profile between US and SS, the sample collection method was a spitting method^17^ (used in this study), whereas the other study used a paper pointing for unstimulated saliva collection^18^. Therefore, the method used for the collection of unstimulated saliva may affect the oral microbial profile.

MW is useful as a method for collecting oral samples from participants who have difficulty collecting saliva, such as dry mouse patients and elderly people. However, in order to prove the usefulness of MW for these participants, its evaluation for participants with low saliva flow, such as individuals who suffer with a dry mouth, is also necessary.

In conclusion, we have shown that the oral microbiome of MW is similar to that of US and SS. Since previous researchers have reported that the salivary microbiome profile can reflect oral and whole-body systematic diseases^13,19,20^, MW is expected to become a beneficial tool for easy evaluation of various disease risks.

## Methods

### Ethics statement

This study was given ethical approval by the ethics committee of Chiyoda Para Medical Care Clinic (Tokyo, Japan, Issuing number: UMIN000031334). All participants understood the purpose of the study and provided informed consent. All experiments were performed in accordance with approved guidelines.

### Sample collection and dental examination

Three male and seven female systemic healthy volunteers aged 24 to 40 years (mean ± s.d., 31.1 ± 4.8 years) were recruited at the Hiyoshi Oral Health Clinics. All participants met the inclusion and exclusion criteria. Inclusion criteria were: (a) non-smoker, (b) non-denture wearer, and (c) non-brace wearer. Exclusion criteria were: (a) systemic disease, (b) received antibiotics in the last 6 months, and (c) pregnancy or breastfeeding.

Sample collection and dental examinations was conducted at the Hiyoshi Oral Health Clinics. Prior to sample collection, the participants were instructed not to brush their teeth from the previous night to the time of sampling, and were prohibited from eating or drinking for at least 1 hour prior to sampling. A minimum of 2 mL unstimulated saliva was collected by allowing saliva to accumulate on the floor of the mouth followed by spitting into a specimen tube every 60 seconds^21^. Following the collection of unstimulated saliva, participants were asked to swish their mouth vigorously for 10 seconds with 3 mL sterilized water, and then to spit into another specimen tube. Following the collection of this mouth-rinsed water, individuals were asked to chew paraffin gum to stimulate saliva, samples of which were then collected in further specimen tubes every 60 seconds. Tongue-coating samples were collected by scraping the dorsum of the tongue with Catch-All™ Specimen Collection Swabs^22^ (Epicenter Biotechnologies, Wisconsin, USA). All samples were placed in a freezer within 10 minutes of collection and stored at −80 °C until use.

After samples collection, the numbers of present, decayed, missing and filled teeth were examined. The number of decayed, missing and filled teeth signifies teeth with caries experience, and represents the caries history of the individual. The periodontal pocket depths and bleeding on probing (BOP) at four sites (mesiobuccal, distobuccal, mesiolingual, and distolingual) of all teeth were measured using a periodontal pocket probe following sample collection. Summary of the oral health condition of the participants is shown in Table 2.

**Table 2:** Summary of participants' information.

Sample ID	B01	B02	B03	B04	B05	B06	B07	B08	B09	B10
Age	40	39	29	28	30	24	27	32	33	29
Gender	M	F	F	F	F	F	F	M	F	M
DMFT	5	0	4	0	0	0	1	20	9	1
Flow rate of Unstimulated Saliva (g/min)	0.86	0.6	1.04	0.54	0.89	0.22	0.62	1.32	0.37	0.69
Flow rate of Stimulated Saliva (g/min)	4.33	3.09	2.75	2.37	4.81	1.45	2.75	3.71	2.73	3.45
PPD > 4 mm (%)	0	1.8	0	2.7	0	0	0	4.3	7.4	0.8
BOP (%)	2.7	15.2	6.3	50.9	2.7	3.6	0	0.9	24.1	2.5

### DNA extraction and sequencing of 16S rRNA gene amplicons

Genomic DNA was isolated from the collected samples using the nexttec™ 1-Step DNA Isolation Kit (nexttec Biotechnologie GmbH, Leverkusen, Germany). PCR was conducted using universal primers (27Fmod and 338R) for 16S rRNA gene sequencing, as previously described^6^. PCR was performed using Ex Taq polymerase (Takara Bio, Shiga, Japan) and approximately 20 ng of template DNA.

Thermal cycling was performed in a Veriti Thermal Cycler (Life Technologies Japan, Tokyo) with the following cycling conditions: initial denaturation at 96 °C for 2 min, followed by 25 cycles of denaturation at 96 °C for 30 s, annealing at 55 °C for 45 s, extension at 72 °C for 1 min, and final extension at 72 °C. PCR amplicons were purified using AMPure XP magnetic purification beads (Beckman Coulter, Brea, CA, USA) and quantified using the Quant-iT PicoGreen dsDNA Assay Kit (Life Technologies Japan). After quantification, mixed samples were prepared by pooling approximately equal amounts of each amplified DNA and sequenced using MiSeq Reagent Kit V3 (300 × 2 cycles) and a MiSeq sequencer (Illumina, CA, USA), according to the manufacturer’s instructions.

### Sequence analysis

The sequenced paired-end reads were merged using VSEARCH (version: 1.9.3, options: -fastq_maxee 9.0, -fastq_truncqual 7, -fastq_maxdiffs 300, -fastq_maxmergelen 450, -fastq_minmergelen 250)^23^. Merged fragments were processed with Cutadapt (version: 1.16, options: -O 13 -m 50 -M 450 -q 0 -e option is not used in cut 5′ primer, used 0.3 in cut 3′ primer) and Bowtie2 (version: 2.1.0, options: -fast-local) for trimming the primer sequences and filtering PhiX fragments, respectively^24,25^. To ensure the quality of the processing reads, fragments with an average Phred quality score of less than 25 were removed using an in-house script. From the filter-passed fragments, 10,000 fragments were randomly subsampled from each sample to remove the effect of sequence-depth bias and then used for the following analysis. The selected fragments were primary dereplicated to remove redundancy, then the dereplicated fragments were clustered into operational taxonomic units (OTUs) using VSEARCH (version: 2.9.1, options:–id 0.99–strand plus). For determining genus/species level taxonomies, the OTUs were mapped onto a reference sequence database using Bowtie2 (version: 2.1.0, options: -I 280 -X 400–fr–no-discordant–phred33 -D 15 -R 10 -N 0 -L 22 -i S,1,1.15). We originally prepared the database by merging the SILVA LTP database (version: 132) and the HOMD 16 S rRNA RefSeq database (version: 15.1)^26,27^. If the edit distance of assigned taxonomy was more than 1% in species assignment or more than 3% in genus assignment, they were assigned as “Unclassified”.

### Statistical analysis

Alpha diversity was measured by the number of OTUs and the Shannon diversity index, which was calculated using an in-house Python script. Beta diversity was measured by Spearman’s rank correlation distance and UniFrac distance, which were calculated using SciPy (version: 0.17.0) and scikit-bio (version: 0.5.2), respectively. An input phylogenetic tree for calculating UniFrac distances was prepared as follows. Multiple sequence alignment of clustered OTUs was performed using MAFFT (version: 7.245) and then a phylogenetic tree was constructed using FastTree (version: 2.1.8), followed by the addition of a midpoint by scikit-bio^28,29^. For the comparison of relative abundance in terms of genera and species, the Friedman test (a non-parametric test for multiple comparisons) and the Benjamini-Hochberg false discovery rate correction (FDR-BH) for posterior correction were adopted. The Friedman test and the Nemenyi post-hoc test were performed using the R package PMCMR (version 4.2, available at https://cran.r-project.org/web/packages/PMCMR/index.html).

### Nucleotide sequence accession number

The microbiome analysis data (16S rRNA gene sequences) have been deposited in the DNA Data Bank of Japan (DDBJ) Sequence Read Archive under accession number DRA008586).

## Supplementary information

Supplementary Table and Figure

Supplementary Table 3

Supplementary Table 4
