# Supplementary material for: Comparison of oral microbiome profiles in stimulated and unstimulated saliva, tongue, and mouth-rinsed water
Source: Sci Rep. 2019 Nov 6;9:16124. doi: 10.1038/s41598-019-52445-6 (PMC6834574; doi:10.1038/s41598-019-52445-6)
Supplement: Supplementary file 1 — Supplementary Table and Figure [file 41598_2019_52445_MOESM1_ESM.docx]

**Comparison of oral microbiome profiles in stimulated and unstimulated saliva, tongue, and mouth-rinsed water.**

Ryutaro Jo^1^, Yuichiro Nishimoto^2^, Kouta Umezawa^3^, Kazuma Yama^4^, Yuto Aita^4^, Yuko Ichiba^4^, Shinnosuke Murakami^2,5^, Yasushi Kakizawa^4^, Takashi Kumagai^3^, Takuji Yamada^2,6,7^, Shinji Fukuda^2,5,7-9*^

1. Oral Care Research Laboratories, Research and Development Headquarter, Lion Corporation. 7-2-1 Hirai, Edogawa-ku, Tokyo 132-0035, Japan.

2. Metabologenomics, Inc. 246-2 Mizukami, Kakuganji, Tsuruoka, Yamagata 997-0052, Japan.

3. Hiyoshi Oral Health Clinics. 2-1-16 Hiyoshi-cho, Sakata, Yamagata 998-0037, Japan.

4. Advanced Analytical Science Research Laboratories, Research and Development Headquarters, Lion Corporation. 7-2-1 Hirai , Edogawa-ku, Tokyo 132-0035, Japan.

5. Institute for Advanced Biosciences, Keio University, 246-2 Kakuganji, Tsuruoka, Yamagata 997-0052, Japan,

6. Department of Life Science and Technology, Tokyo Institute of Technology, 2-12-1 Ookayama, Meguro, Tokyo 152-8550, Japan,

7. PRESTO, Japan Science and Technology Agency, 4-1-8 Honcho Kawaguchi, Saitama 332-0012, Japan

8. Intestinal Microbiota Project, Kanagawa Institute of Industrial Science and Technology, 3-25-13 Tonomachi, Kawasaki-ku, Kawasaki, Kanagawa 210-0821, Japan

9. Transborder Medical Research Center, University of Tsukuba, 1-1-1 Tennodai, Tsukuba, Ibaraki 305-8575, Japan

*Correspondence:

Shinji Fukuda

Metabologenomics, Inc.

246-2 Mizukami, Kakuganji, Tsuruoka, Yamagata 997-0052, Japan.

E-mail: sfukuda@metagen.co.jp

TEL: (+81) 235-64-0330

Supplementary Table S1. Spearman Rank Correlation between mouth-rinsed　water, unstimulated saliva, stimulated saliva（mean ± standard deviation）. SS, stimulated saliva; US, unstimulated saliva; MW, mouth-rinsed water.

|  | MW | SS | US |
| --- | --- | --- | --- |
| SS | 0.858 ± 0.038 |  |  |
| US | 0.862 ± 0.052 | 0.846 ± 0.033 |  |
| TC | 0.744 ± 0.081 | 0.776 ± 0.080 | 0.747 ± 0.080 |

Supplementary Table S2.　Spearman Rank Correlation between mouth-rinsed water, unstimulated saliva, stimulated saliva for each subject.

MW, mouth-rinsed water; US, unstimulated saliva; SS, stimulated saliva.

|  | B01 | B02 | B03 | B04 | B05 | B06 | B07 | B08 | B09 | B10 |
| --- | --- | --- | --- | --- | --- | --- | --- | --- | --- | --- |
| US-MW | 0.870 | 0.899 | 0.848 | 0.908 | 0.910 | 0.858 | 0.920 | 0.846 | 0.826 | 0.736 |
| SS-MW | 0.852 | 0.846 | 0.842 | 0.889 | 0.896 | 0.894 | 0.910 | 0.849 | 0.825 | 0.778 |
| TC-MW | 0.591 | 0.812 | 0.712 | 0.750 | 0.636 | 0.842 | 0.850 | 0.772 | 0.779 | 0.699 |

Supplementary Table S3. Bacterial composition of each samples at genus level.

Supplementary Table S4. Bacterial composition of each samples at species level.

(Please refer to attached Excel File)


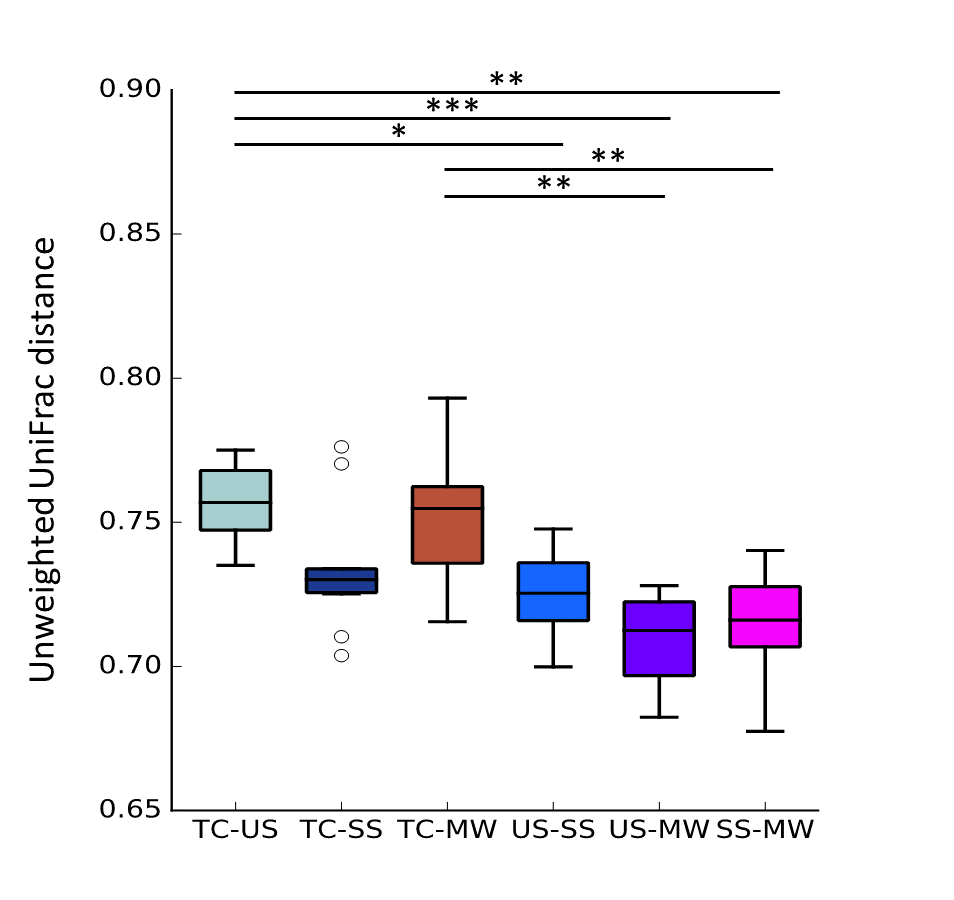


Supplementary Figure S1A


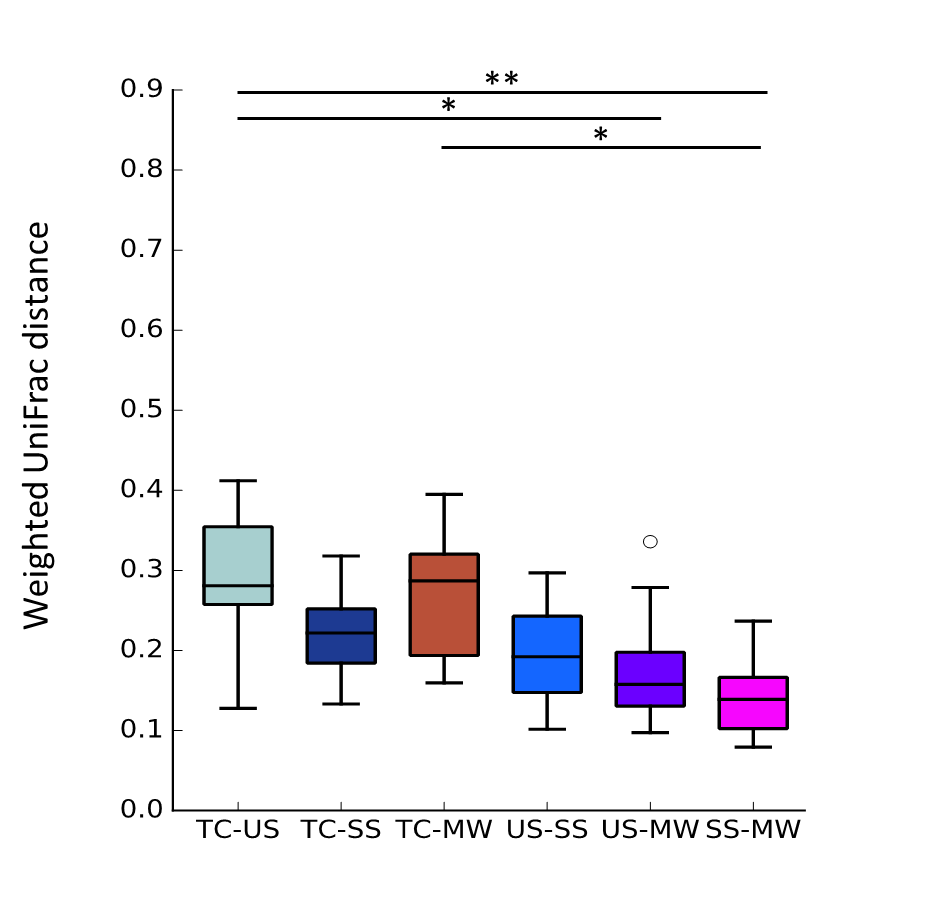


Supplementary Figure S1B

Supplementary Figure S1. Unweighted (A) and Weighted (B) UniFrac distance between each sampling method. TC, Tongue coating; US, unstimulated saliva; SS, stimulated saliva; MW, mouth-rinsed water. Statistically significant differences are marked with asterisks (Nemenyi test, *, p < 0.05　**, p < 0.005　***, p < 0.0005)
